# Delayed plasma kallikrein inhibition fosters post-stroke recovery by reducing thrombo-inflammation

**DOI:** 10.1186/s12974-024-03149-w

**Published:** 2024-06-13

**Authors:** Steffen Haupeltshofer, Stine Mencl, Rebecca D. Szepanowski, Christina Hansmann, Ana I. Casas, Hanna Abberger, Wiebke Hansen, Alina Blusch, Cornelius Deuschl, Michael Forsting, Dirk M. Hermann, Friederike Langhauser, Christoph Kleinschnitz

**Affiliations:** 1 Department of Neurology and Center for Translational Neuro- and Behavioral Sciences (C-TNBS), University Hospital Essen, Hufelandstr. 55, D-45147 Essen, Germany; 2 Department of Pharmacology & Personalized Medicine, MeHNS, Faculty of Health, Medicine & Life Science, Maastricht University, Maastricht, The Netherlands; 3 Institute of Medical Microbiology, University Hospital Essen, Virchowstr. 179, D-45147 Essen, Germany; 4 Division of Immunology, Walter and Eliza Hall Institute of Medical Research, Parkville, VIC 3052 Australia; 5 Department of Medical Biology, University of Melbourne, Parkville, VIC 3052 Australia; 6 Department of Neurology, Center for Huntington’s Disease NRW, St. Josef-Hospital, Ruhr-University Bochum, Gudrunstr. 56, D-44791 Bochum, Germany; 7 Institute of Diagnostic and Interventional Radiology and Neuroradiology, University Hospital Essen, Hufelandstr. 55, D-45147 Essen, Germany; 8 Chair of Vascular Neurology, Dementia and Ageing, Department of Neurology, Medical Research Centre, University Hospital Essen, Hufelandstr. 55, D-45147 Essen, Germany

**Keywords:** Thrombo-inflammation, Thrombosis, Ischemic stroke, Recovery, Subacute, Plasma kallikrein, Kallikrein-kinin system, Blood-brain barrier, Extravasation, Inflammation

## Abstract

Activation of the kallikrein-kinin system promotes vascular leakage, inflammation, and neurodegeneration in ischemic stroke. Inhibition of plasma kallikrein (PK) – a key component of the KKS – in the acute phase of ischemic stroke has been reported to reduce thrombosis, inflammation, and damage to the blood-brain barrier. However, the role of PK during the recovery phase after cerebral ischemia is unknown. To this end, we evaluated the effect of subacute PK inhibition starting from day 3 on the recovery process after transient middle artery occlusion (tMCAO). Our study demonstrated a protective effect of PK inhibition by reducing infarct volume and improving functional outcome at day 7 after tMCAO. In addition, we observed reduced thrombus formation in cerebral microvessels, fewer infiltrated immune cells, and an improvement in blood-brain barrier integrity. This protective effect was facilitated by promoting tight junction reintegration, reducing detrimental matrix metalloproteinases, and upregulating regenerative angiogenic markers. Our findings suggest that PK inhibition in the subacute phase might be a promising approach to accelerate the post-stroke recovery process.

**Supplementary Information:**

The online version contains supplementary material available at 10.1186/s12974-024-03149-w.

## Background

Ischemic stroke is a prevalent neurovascular event, impacting more than 60 million patients per year worldwide [1]. Currently, the only available treatments for cerebral ischemia are thrombolysis with tissue plasminogen activator or mechanical thrombectomy [2, 3]. However, these treatments are restricted to a short time window in the acute phase after stroke; currently, no treatment options support the recovery phase after stroke.

Following ischemic stroke, thrombus formation and inflammation are closely connected, a phenomenon referred to as thrombo-inflammation [4]. The kallikrein-kinin system (KKS) represents an interface between thrombotic and inflammatory circuits and consists of serially connected serine proteases, initiated by the activation of factor XII (FXIIa). Specifically, FXIIa activates plasma kallikrein (PK) leading to cleavage of high molecular weight kininogen and the release of the proinflammatory peptide hormone bradykinin, thus fostering disruption of the blood-brain barrier (BBB) and cerebral inflammation [4].

Previous investigations have suggested that inhibition of KKS components improves stroke outcomes and decreases infarct volumes in acute ischemic stroke [5]. Factor XII inhibition and its genetic depletion mediated ischemic stroke protection with beneficial neurological outcomes, without increasing the intracranial bleeding risk [6, 7]. Similarly, mice with genetic depletion of kininogen showed less susceptibility to post-ischemic brain damage by a reduction in the inflammatory response, less microvascular thrombosis, and improved BBB integrity [8]). Acute PK inhibition or genetic depletion of PK also mediated protection in experimental ischemic stroke by reducing infarct size and inflammation and enhancing cerebral blood flow [9]. However, these studies addressed KKS inhibition in the acute phase of cerebral ischemia starting at the time of reperfusion or in genetic depletion models. So far, the impact of delayed KKS inhibition on stroke recovery has not been evaluated.

To this end, we investigated the effect of PK inhibition in the subacute phase after ischemic stroke in mice subjected to transient middle cerebral artery occlusion (tMCAO). Our aim was to evaluate the effects of delayed treatment with αPK antibodies (starting from day 3 post-stroke) on neurological recovery, thrombosis, inflammation, and BBB integrity.

## Methods

### Experimental animals

All experiments were conducted in accordance with the IMPROVE guidelines for animal experimentation [10] and reported based on ARRIVE guidelines [11]. The study was approved by the regional government authorities (Landesamt für Natur, Umwelt und Verbraucherschutz NRW, LANUV) and guided by the German Welfare Act (German Ministry of Agriculture, Health, and Economic Cooperation). Male C57BL/6J mice were purchased from Charles River (Sulzfeld, Germany) and housed in a temperature- and humidity-controlled, specific pathogen-free animal facility with a 12-h light-dark cycle, as well as food and water available *ad libitum*. Mice were randomly assigned to experimental groups by investigators not involved in the data acquisition or analysis. All efforts were made to minimize animal suffering and the number of animals used. Surgeries, treatments, and data analyses were performed by investigators blinded to the experimental animals. Experimental analyses were mainly limited to male mice for reasons of consistency with previous studies, in which we evaluated the effects of PK inhibition on ischemic injury and neurological outcome in the acute stroke phase in male mice [9].

### Ischemia model and neurological assessment

In 10-12-week-old mice, focal cerebral ischemia was induced by tMCAO for 60 min occlusion time [12]. Mice were anesthetized with 4% isoflurane (Piramal) in 100% oxygen for 3–5 min (World Precision Instruments, Small Animal Anesthesia System, EZ-7000). Anesthesia was maintained with ∼ 2% isoflurane, and body temperatures were kept at 37 °C during surgery, using a feedback-controlled warming device (World Precision Instruments, Small Animal Anesthesia System, EZ-7000). Occlusion of the middle cerebral artery (MCA) was induced as described [12]. In brief, the filament (#602112PK5Re, Doccol) was introduced to the MCA via the external carotid artery with a reopening of transient ligation at the common carotid artery to ensure full reperfusion. Cerebral blood flow was measured for successful occlusion and reperfusion using Laser Doppler flowmetry (PeriFlux 6000, Perimed, Järfälla, Sweden). Neurological deficits following tMCAO were measured utilizing a modified version of the neuroscore [13]. This score is composed of a global and focal deficit subscore. Points covered by the global score include spontaneous activity and fur abnormalities, while focal deficits encompass body symmetry, gait circling behavior, symmetry of paws and body, and whisker response. In this study, we used the combined score. Motor strength, sensorimotor coordination, and endurance were measured using the rotarod (RR) test (Rotarod Advanced for Mice, TSE Systems, Germany) [14], adhesive removal (AR) test [14], and the tightrope (TR) test [15], which were performed as recently described by our group [16]. Better behavioral results were reflected by higher point or time scores in the RR and TR tests. In the AR test and the modified neuroscore, less time or lower point scores were indicative of a better behavioral performance. All analyses were carried out in a day 3 and day 7 time frame after tMCAO.

### MRI scanning and analyses

To analyze infarct volume and identify intracerebral bleeding, we performed serial stroke assessment using magnetic resonance imaging (MRI) with a 3 Tesla MRI unit (Biograph mMR, Siemens Healthineers, Erlangen/Germany) on days 3 and 7 after tMCAO. We used dual channel surface coils designed for animal experimentation (Rapid Biomedical GmbH, Rimpar/Germany). The imaging protocol included a coronal T2-weighted, turbo spin-echo (TSE) sequence (resolution 0.2 × 0.2 × 0.8 mm³, 15 slices, TE = 105 ms, TR = 1660 ms, TA = 3:47 min) to identify infarct volume, and a susceptibility-weighted, coronal T2-weighted gradient echo sequence (SWI, 0.1 × 0.1 × 0.2 mm³, 40 slices, TE = 20.6 ms, TR = 38 ms, TA = 6:01 min) to detect hemorrhage. Refined localization was done with the help of short T1-weighted TSE sequences along each axis (0.2 × 0.2 × 1 mm³, seven slices, TA = 17 s). Brain edema and infarct were measured from MRI coronal T2 images. Percentage brain edema was calculated based on the following equation: ([ipsilateral hemisphere volume – contralateral hemisphere volume]/[contralateral hemisphere volume]) × 100. Edema was calculated as follows: ([contralateral hemisphere volume] – [ipsilateral hemisphere volume]) x100. By multiplying the area of tissue loss by the distances between selected brain sections, brain infarct volume and volume of tissue loss was determined. Only mice without bleeding and comparable infarct volume between 50 and 70 mm^3^ on day 3 were included in the experiment. Infarct sizes were additionally visualized using triphenyl tetrazolium chloride (TTC) and hematoxylin and eosin (H&E) staining. Of 63 mice with tMCAO, 54 were included in the final analysis (*n* = 18 each group). 9 mice were excluded due to either hemorrhagic transformation or inconsistent infarct sizes.

### Pharmacological intervention

Based on the MRI infarct volume calculation on day 3, animals with comparable infarct sizes (50–70 mm^3^) were included for further experimentation. Animals were randomly assigned to treatment groups from outside assistance not involved in data acquisition or analysis. Antibodies to block PK (αPK, Ab1006, Abcam) or its corresponding isotype control (immunoglobulin G (IgG); BioLegend) were administered intravenously (tail vein) as follows: 400 µg/kg on days 3 and 4 and 200 µg/kg at days 5 to 7 after stroke induction. The antibody concentration was based on our previous study of PK inhibition in the acute phase of ischemic stroke [9].

### Enzyme-linked immunosorbent assay (ELISA)

To investigate activation of the KKS after tMCAO in respective treatment conditions, we measured serum levels of PK (Arigo ARG82751) and bradykinin (Phoenix Pharmaceuticals, EK-009-01) and proceeded as described in the manufacturer’s protocol. Inflammatory cytokines (IL-1β, IL-6, RANTES, MCP-1, MCP-2, GM-CSF, TNFα), degradation markers (MMP-2, MMP-9) and angiogenic markers (VEGF, PDGFb) were measured using R&D Systems customized LUMINEX plates. Samples were processed according to the manufacturer’s protocol, measured with a MAGPIX Luminex device and the data analyzed with the Luminex xPONENT software.

### Flow cytometry

Animals were euthanized and perfused with cold saline. Brains were dissected into the ipsilateral (left) and contralateral (right) hemisphere. Immune cells were isolated using the Neural Tissue Dissociation Kit™ (Miltenyi Biotec) and a gentleMACS dissociator with heaters (Miltenyi Biotec), following the manufacturer’s instructions. Fluorochrome compensation was performed with BD Diva integrated compensation matrix and single-stained, mixed-tissue controls. Flow cytometric analysis was performed on the FACSAria III flow cytometer (BD Bioscience). Data analyses were performed using FlowJo software. Antibodies were used as following: anti-CD3 PacBlue (100,214, Biolegend, 1:400), anti-CD45-HorizonV500 (561,487, Biolegend, 1:400), eFluor 780 Viability dye (103,210, Thermo Fisher, 1:400), anti-CD11b-APC (101,212, Biolegend, 1:400), and anti-Ly6G-PerCP-Cy5.5 (127,615, Biolegend, 1:400).

### Histological and western blot analyses

Coronal brain sections were subjected to immunofluorescence staining as described previously [16]. The following primary antibodies were used for histological stainings: anti-PK (αPK; ab1006, Abcam, 1:200), anti-zonula occludens (ZO)-1 (ab216880, Abcam, 1:200), anti-Claudin-5 (ab131259, Abcam, 1:200), anti-occludin (ab216327, Abcam, 1:200), anti-CD31 (mca2388, BioRad, 1:100), anti-GPIX (M052-0, Emfret, 1:100), anti-albumin (ab207327, Abcam, 1:200), anti-bradykinin-1 receptor (B1R; ABR-011, Alomone Labs, 1:200), anti-CD11b (MCA74G, Biorad, 1:200, unfixed tissue) and anti-NG2 (ab5320, Millipore, 1:200). All secondary antibodies were diluted 1:1000. Fluorescence stainings were visualized using a Leica DMi8 microscope, Hamamatsu C11440-22 CU camera, and Leica Application Software X (LasX 3.0.2.16120). Images were processed using Image J (National Institutes of Health). Western blot analyses using anti-GP1bα (M043-0, Emfret, 1:200) was performed as previously described [9].

### Quantitative real-time PCR analyses

To analyze transcriptional changes in the brain or purified endothelial cells from tMCAO animals, real-time polymerase chain reaction (PCR) was performed. The procedure of using the SYBR green system (Promega, A6001) was as previously described [17] and processing took place on a QuantStudio 3 RT-PCR machine (Life Technologies). In brief, endothelial cells were isolated from the ipsilateral hemisphere of tMCAO mice using Kit1 for dissociation of inflamed neural tissue (Miltenyi Biotec, #130-110-201). Endothelial cells were then purified using the CD31 MicroBeads isolation kit (Miltenyi Biotec, #130-097-418). For both the whole brain sections and the purified cells, mRNA was isolated (RNeasy MicroKit, Qiagen, 74,004) and converted into cDNA (QuantiTect Reverse Transcription Kit, Qiagen, 205,311), as per the manufacturer’s protocol. The following sequence-specific sense (sen) and antisense (ase) primers were designed to measure mRNA expression levels: arginase 1 (Arg-1; Fwd CAT TCT TCG CTG CCA TTC TG; Rev GCA CAT TGC CCA TGT TGA ATC), Ras-related C3 botulinum toxin substrate 1 (Rac1; Fwd GGG GAT CCC AGG CCA TCA AGT GTG TGG TGG; Rev GGA ATT CTT ACA ACA GCA GCA GGC ATT TTC TCT TCC), vascular endothelial growth factor (VEGF; Fwd GGA GAT CCT TCG AGG AGC ACT T; Rev GGC GAT TTA GCA GCA GAT ATA AGA A), Rho-associated kinases (ROCK; Fwd AAC ATG CTG CTG GAT AAA TCT GG; Rev TGT ATC ACA TCG TAC CAT CCT), alpha-5 integrin (α5; Fwd TGT CAC CGT CCT TAA TGG; Rev CAT TGT AGC CGT CTT GGT), β-actin (Fwd CCA AGG CCA ACC GCG AGA AGA TGA C; Rev AGG GTA CAT GGT GGT GCC GCC AGA C), and glyceraldehyde-3-phosphate dehydrogenase (GAPDH; Fwd AGG TCG GTG TGA ACG GAT TTG; Rev TGT AGA CCA TGT AGT TGA GGT C). Relative target mRNA expression was normalized to the geometric expression average of the housekeeping genes β-actin and GAPDH. We applied each sample in two technical replicates for each data point. The mean cycle threshold (Ct) was used in the equation for the housekeeping genes and Ct for the genes of interest.

### Statistical analysis

Sample sizes for animal studies were determined using power analyses, allowing us to detect differences with 80% power at the expected effect sizes and with the expected variance observed in that assay. The expected effect size was 0.5. Power analyses were primarily based on our previous work in preclinical stroke [6–9]. The type I error rate was limited using an α level of 5%, with Bonferroni’s adjustment for pairwise comparisons. Results are presented as mean ± standard deviation. Of note, explanatory schematics were created with biorender. GraphPad Prism software (v9.0.0) was used for statistical analyses and visualization of the results. To evaluate the normal distribution of the data sets, we performed the D’Agostino-Pearson test. The 2-tailed Student’s t-test was used for normally distributed data if applicable; otherwise, we used the Mann-Whitney U test for non-normally distributed datasets. The differences among multiple groups were analyzed using 1-way or 2-way ANOVA or non-parametric Kruskal-Wallis tests as appropriate. Differences between groups were assessed with post hoc Bonferroni (comparisons between all conditions), Dunnett (all conditions compared with a control group), or Dunn’s (following Kruskal-Wallis) tests. In all analyses, a *P*-value ≤ 0.05 was considered statistically significant.

## Results

### Inhibition of PK in the subacute phase reduced infarct volume and improved neurological recovery after ischemic stroke

**Fig. 1:**
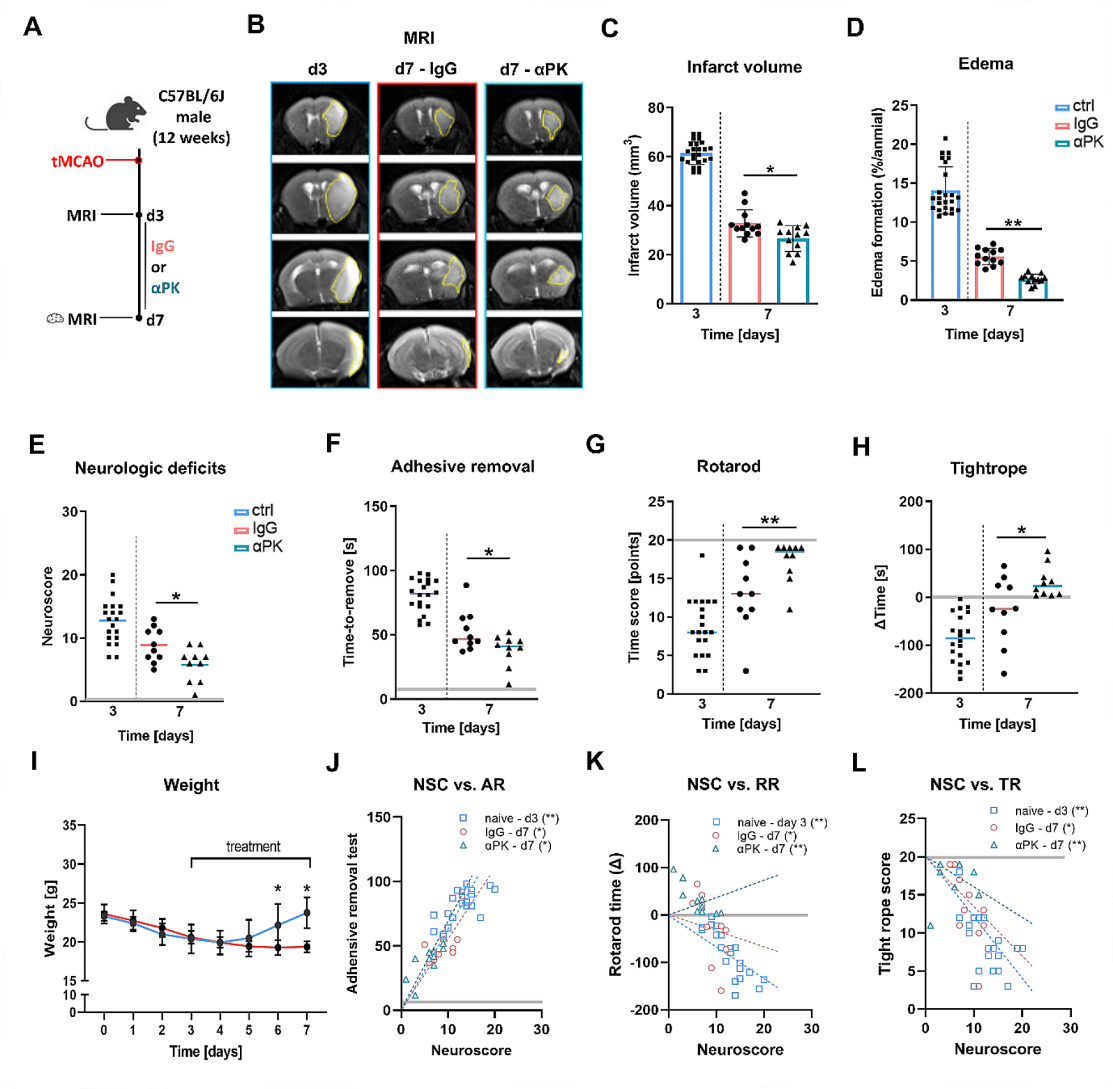
Delayed anti-plasma kallikrein (αPK) treatment transiently improved focal deficits and reduced edema formation. (**A**) Mice were treated with isotype immunoglobulin G (IgG) or αPK at days 3 and 4 (400 µg/kg) and days 5 to 7 (200 µg/kg) after 60-min transient middle artery occlusion (tMCAO). Cerebral damage was visualized using magnetic resonance imaging (MRI) at days 3 and 7. (**B**) Representative images of T2 MRI scans 3 and 7 days after tMCAO. The yellow dotted lines depict infarct areas. Quantification of (**C**) brain infarct volume and (**D**) brain edema (*n* = 24/12/12). (**E**) Assessment of general and focal deficits at 3 and 7 days of tMCAO mice treated with IgG or αPK. Evaluation of sensorimotor function of tMCAO with (F) adhesive removal test (AR), (**G**) rotarod test (RR), and (**H**) tightrope test (TR) (*n* = 20/10/10). (**I**) Quantification of body weight at days 0 to 7 after tMCAO (TR) (*n* = 10/10). (**J**–**L**) Spearman correlation of sensorimotor deficits with neurological score evaluation. Two-way ANOVA and post hoc Sídák test. *P < 0.05, **P < 0.01, ***P < 0.001 for αPK vs. IgG; grey lines represent the baseline values measured in healthy mice

Inhibition of PK in the acute ischemic stroke has been shown to reduce infarct volumes and improve neurological outcome [9]. Therefore, we investigated whether inhibition of PK in the subacute phase is also protective. C57BL/6J mice were subjected to 60-min tMCAO and after complete infarct maturation on day 3, magnetic resonance imaging (MRI) was performed to evaluate infarct volume. Additionally, successful occlusion of the MCA and reperfusion was monitored using Laser Doppler flowmetry (Suppl. Figure 1). To ensure comparable baseline conditions, only animals with similar infarct sizes were randomly assigned for treatment with either αPK (at doses of 400 µg/kg on days 3 and 4, followed by 200 µg/kg on days 5–7) or IgG as control (Fig. 1A + B; Suppl. Figure 2). Quantitative analysis of infarct volumes and edema formation via MRI demonstrated a significant reduction in animals treated with αPK compared to controls at day 7 post-stroke (Fig. 1C + D). The reduction in infarct size was associated with markedly enhanced functional outcomes, as evidenced by modified neuroscore assessments (Fig. 1E). Detailed functional evaluations encompassing sensorimotor abilities, including AR, RR, and TR, revealed substantial improvement in αPK-treated mice relative to controls at day 7 after tMCAO (Fig. 1F–H). Notably, αPK-treated mice exhibited consistent weight gain from day 5 onwards, indicative of progressive functional recovery (Fig. 1I). Correlation analyses further underscored a significant relationship between reduced neurological deficits and improved functional outcomes in αPK-treated mice versus controls at both day 3 and day 7 post-stroke (Fig. J–L).

### Delayed PK inhibition reduced thrombo-inflammation after brain ischemia

**Fig. 2:**
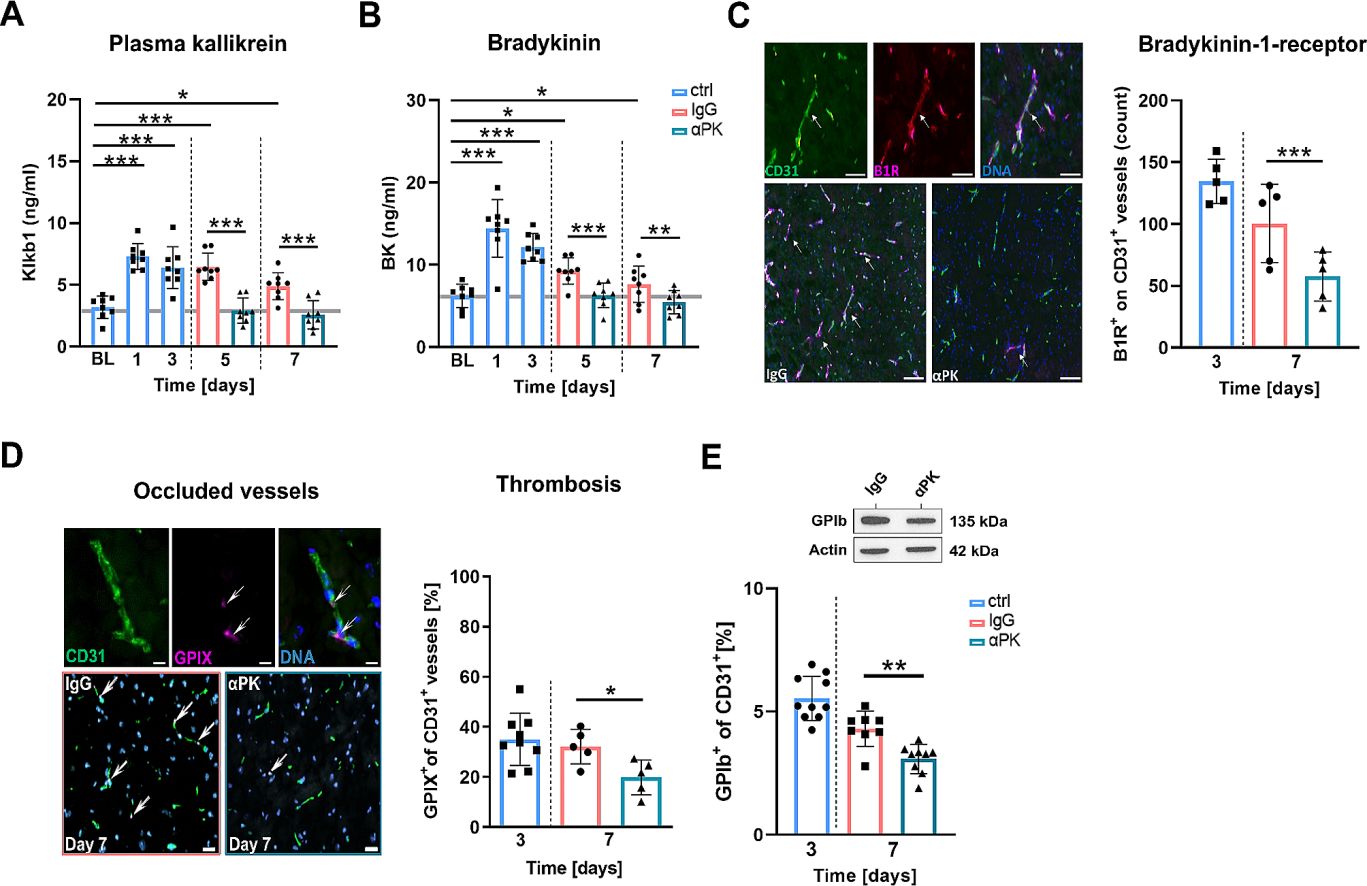
Subacute inhibition of plasma kallikrein (PK) protected against persistent thrombosis in microvasculature transient middle artery occlusion (tMCAO). (**A**) Quantification of activated PK and (**B**) its respective downstream target bradykinin in the blood at day − 1 (baseline) and days 1, 3, 5, and 7 after tMCAO of mice treated with immunoglobulin G (IgG) or anti-PK (αPK) (*n* = 8). (**C**) Histological staining of CD31^+^ blood vessels and the bradykinin-1 receptor (B1R^+^) at days 3 and 7 (white arrows) (*n* = 5). (**D**) Histological staining of CD31^+^ blood vessels and GPIX^+^ platelet adhesion molecules showing thrombi at days 3 and 7 after tMCAO (white arrows) (*n* = 9/5/5). (**E**) Protein density analysis of platelet adhesion molecule GP1b at days 3 and 7 after tMCAO (*n* = 10/8/8). One-way ANOVA and post hoc Dunn’s test. *P < 0.05, **P < 0.01, ***P < 0.001 for αPK vs. IgG

PK has been described as a key mediator of thrombus formation and inflammation after ischemic stroke [9, 18]. To understand the impact of PK on the recovery phase, we determined thrombo-inflammatory consequences of delayed PK inhibition. Administration of αPK (400 µg/kg at day 3 + 4, 200 µg/kg at day 5–7) reduced activated PK and its downstream protein bradykinin in the blood at day 5 and day 7 after cerebral ischemia compared to IgG-treated control mice (Fig. 2A + B). Histological analysis revealed significantly reduced B1R on CD31^+^ vessels in αPK-treated mice at day 7 after tMCAO compared to controls (Fig. 2C). In line with reduced KKS activation, inhibition of PK reduced microvascular thrombus formation at day 7 after tMCAO compared to control mice (Fig. 2D + E).

### PK inhibition reduced BBB breakdown after brain ischemia

**Fig. 3:**
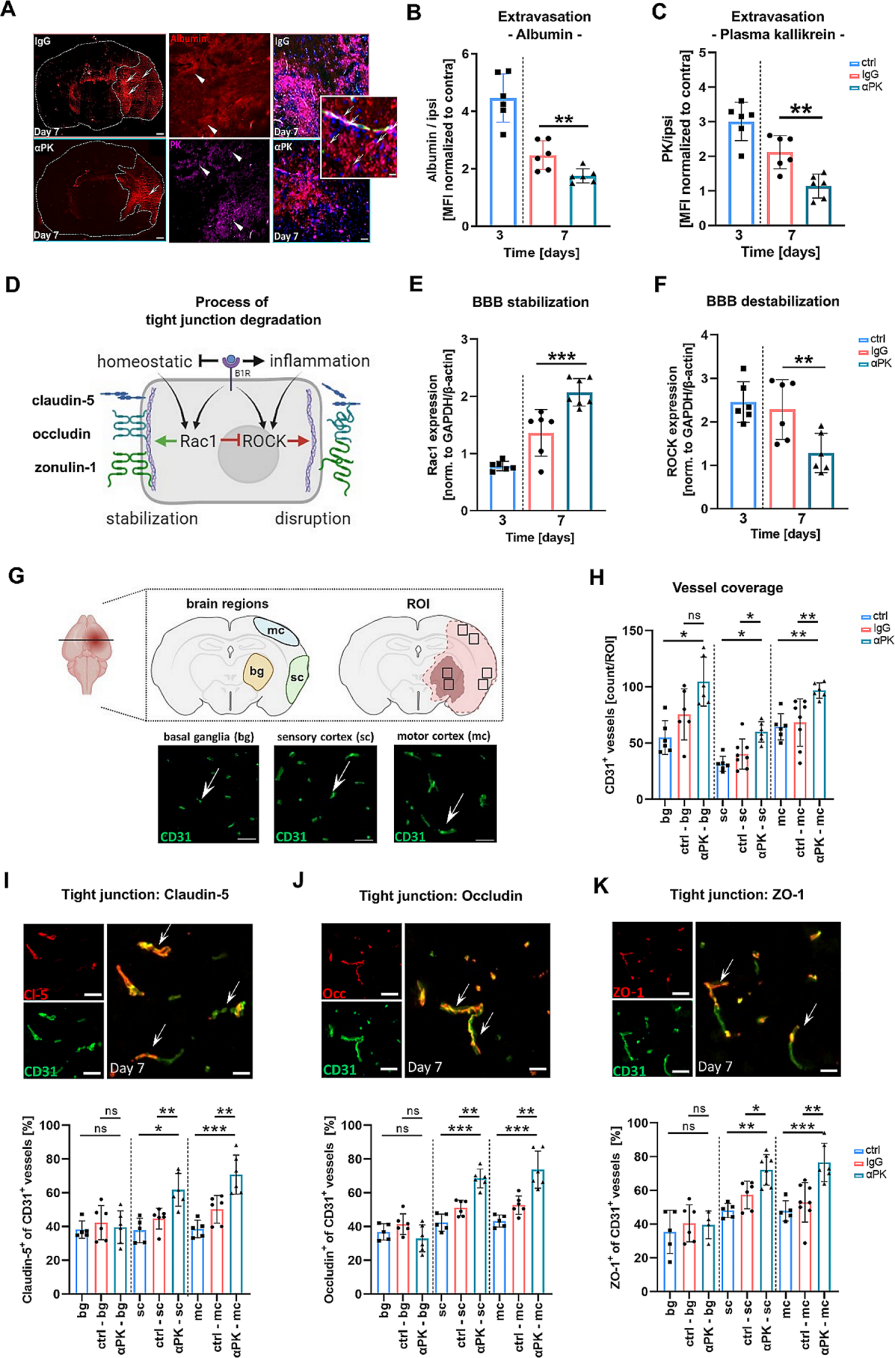
Blockade of plasma kallikrein (PK) was essential for blood-brain barrier (BBB) stabilization and attenuation of extravasation after transient middle artery occlusion (tMCAO). (**A**) Exemplary staining of albumin and PK (white arrows) in the ischemic lesion. Extravasation of (**B**) albumin and (**C**) blood-resident PK into the brain parenchyma of immunoglobulin G (IgG)- and anti-PK (αPK)-treated mice (*n* = 6). (**D**) Schematic pathway of tight junction (TJ) protein degradation in endothelial cells bradykinin-1 receptor (B1R) activation under pathophysiological conditions. (**E**) Ras-related C3 botulinum toxin substrate 1 (Rac1)-axis activation led to stabilization of the BBB (*n* = 8). (**F**) Intracellular Rho-associated kinases (ROCK)-axis activation mediated degradation of the BBB after ischemic stroke (*n* = 8). (**G**) Representative image of anti-CD31 histological staining and determination of region of interest (ROI) in the basal ganglia and sensory and motor cortex. (**H**) Quantitative analysis of histological CD31^+^ vessel density from respective brain regions at days 3 and 7 (IgG vs. αPK) (*n* = 6–8). (**I**–**K**) Representative images of TJ protein staining and analysis of histological staining for CD31 vessel density and co-expression of claudin-5, occludin, and ZO-1 at days 3 and 7 after tMCAO in the respective IgG and αPK groups (*n* = 5–6). One-way ANOVA and post hoc Dunn’s test. *P < 0.05, **P < 0.01, ***P < 0.001 for αPK vs. IgG

After ischemia, the BBB opens within hours after infarction, permitting the influx of blood-borne components and immune cells into the brain [19, 20]. Therefore, we determined whether PK affects the BBB permeability in the subacute phase. Inhibition of PK led to significantly reduced extravasated albumin and PK in the brain parenchyma at day 7 after cerebral ischemia in αPK-treated mice (Fig. 3A–C). As a next step, we determined the underlying mechanisms that stabilized the BBB. Cerebral ischemia induces tight junction (TJ) protein degradation via mechanisms involving activation of the B1R/ROCK/Rac1 pathway [21] (Fig. 3D). Reverse transcription PCR (RT-PCR) analysis revealed significantly increased Rac1 expression (Rac1 stabilized the BBB) and decreased ROCK expression (ROCK-promoted BBB degradation) in purified endothelial cells derived from αPK-treated mice compared to control endothelial cells at day 7 after ischemia (Fig. 3E + F). αPK-treated mice exhibited significantly increased numbers of CD31^+^ blood vessels in the peri-infarct area (sensory and motor cortex), but not in the infarct core (basal ganglia) at day 7 after tMCAO compared to controls (Fig. 3G + H). In addition, αPK treatment significantly increased claudin-5, occludin, and ZO-1 co-localization with vessels in the peri-infarct area, while the infarct core was unaffected at day 7 after stroke compared to control mice (Fig. 3F–H), suggesting stabilization of the BBB by PK blockade in the subacute stroke phase.

### Subacute PK inhibition reduced BBB-permeabilizing proteases and increased angiogenic markers

**Fig. 4:**
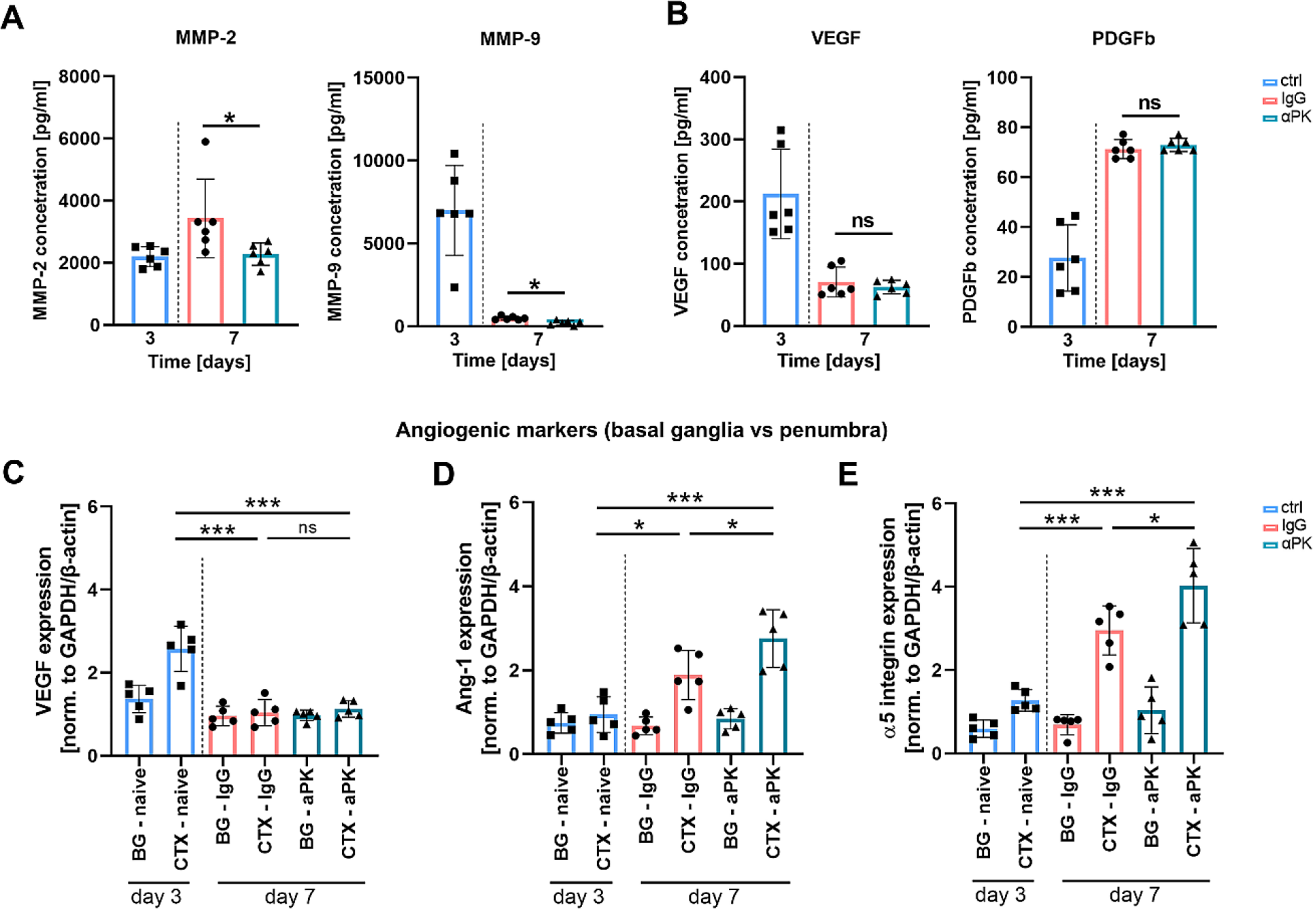
Delayed plasma kallikrein (PK) inhibition blocked destabilization of the blood-brain barrier (BBB), leading to enhanced angiogenic marker expression after transient middle artery occlusion (tMCAO). Secretion patterns on the ipsilateral side of ischemic brains at day 3 and day 7. Amount of disruptive (**A**) matrix metalloproteinases (MMP)-2 and − 9, (**B**) vascular endothelial growth factor (VEGF), and platelet-derived growth factor subunit B (PDGFb) angiogenic markers (*n* = 6). Expression analysis of angiogenic-supporting factors (**C**) VEGF, (**D**) angiopoietin-1 (Ang-1), and (**E**) integrin-5 (α5) subunit from the basal ganglia (BG; infarct core) and cortex (CTX) from ischemic mouse brains (*n* = 5). One-way ANOVA and post hoc Dunn’s test. *P < 0.05, **P < 0.01, ***P < 0.001 for αPK vs. IgG

To elucidate how BBB stabilization was supported by the brain environment, we determined matrix metalloproteinases (MMPs) and angiogenic markers by ELISA. Following PK inhibition, a significant reduction in MMP-2 and MMP-9 was found in brain tissue extracts at day 7 after tMCAO compared to control mice (Fig. 4A), suggesting less extracellular matrix degradation which supports BBB integration. We further elucidated the abundance of angiogenic molecules in the ischemic hemisphere and measured no alterations in VEGF levels and platelet-derived growth factor subunit B (PDGFb) levels at day 7 (Fig. 3B). However, RT-PCR analysis of VEGF (Fig. 4C), angiopoietin-1 (Ang-1) (Fig. 4D), and alpha-5 integrin subunit (Fig. 4E) revealed increased expression of the angiogenic markers in the peri-infarct area, indicating prominent microvascular remodeling in the peri-infarct brain tissue.

### Delayed PK inhibition decreased inflammation during recovery after cerebral ischemia

**Fig. 5:**
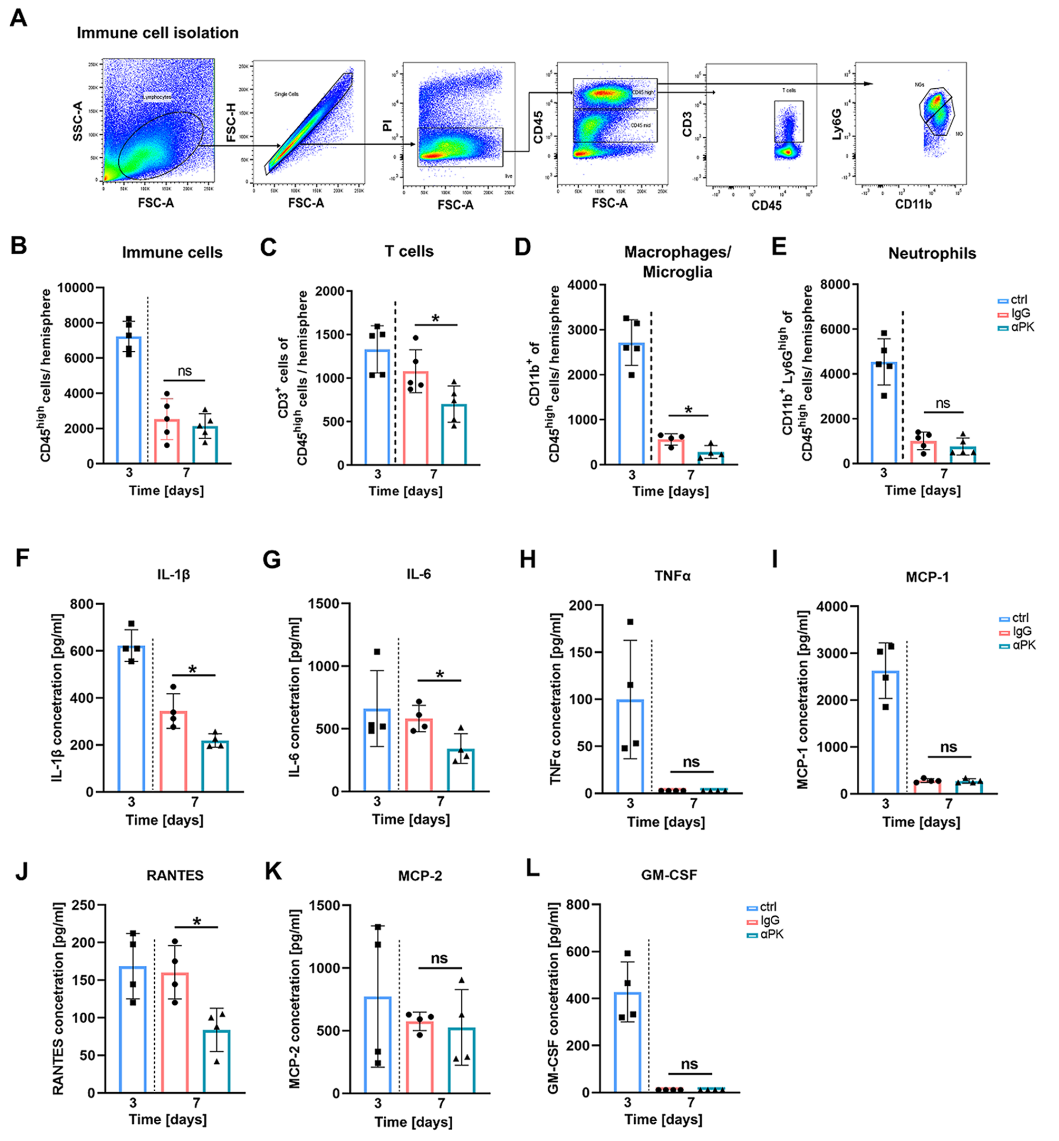
Plasma kallikrein (PK) inhibition induced alteration of immune cell influx and ameliorated inflammatory cytokines after transient middle artery occlusion (tMCAO). (**A** + **B**) Representative dot plot of brain-isolated immune cells and quantitative analysis of infiltrated immune cells into the ischemic brain at days 3 and 7 after immunoglobulin G (IgG) and anti-PK (αPK) in tMCAO mice (*n* = 5). Analysis of (**C**) CD3^+^ lymphocytes (adaptive immune system), (**D**) macrophages/microglia, and (**E**) neutrophil granulocytes (innate immune system) from IgG- and αPK-treated tMCAO mice (*n* = 5). Inflammatory cytokines (**F**) interleukin (IL)-1β, (**G**) IL-6, (**H**) tumor necrosis factor alpha (TNFα), (**I**) monocyte chemoattractant protein (MCP)-1, (**J**) regulated and normal T cell expressed and secreted (RANTES), (**K**) MCP-2, and (**L**) granulocyte-macrophage colony-stimulating factor (GM-CSF) in the brain parenchyma (*n* = 4). One-way ANOVA and post hoc Dunn’s test. *P < 0.05 for αPK vs. IgG

Investigating the influx of immune cells did not yield alterations in overall CD45^high^ leukocytes between treatment groups at day 7 (Fig. 5A + B). Deeper analysis of immune cell composition, however, revealed reduced numbers of CD45^high^CD3^+^ T cells and CD45^high^CD11b^+^ macrophages/microglia in the brains of αPK-treated mice compared to controls at day 7 after ischemia (Fig. 5C + D, Suppl. Figure 3). The number of CD45^high^CD11b^+^Ly6G^+^ neutrophils was unaffected by PK blockade (Fig. 5E). Cytokine analysis of the ischemic hemisphere revealed a pro-inflammatory milieu at day 3 (Fig. 5F). Treatment with ⍺PK reduced the level of interleukin (IL)-1β, IL-6, and RANTES (i.e., regulated and normal T cell expressed and secreted) in tMCAO mice. Granulocyte-macrophage colony-stimulating factor (GM-CSF), tumor necrosis factor alpha (TNFα), and monocyte chemoattractant protein (MCP)-1 and − 2 were highly abundant at day 3 compared to concentrations found at day 7 after tMCAO. However, GM-CSF and TNFα were no longer detectable at day 7, while MCP-1 and − 2 were comparably reduced and not influenced by αPK treatment at this time point (Fig. 5F–L).

## Discussion

We studied the benefits of subacute PK inhibition in a murine model of ischemic stroke. Prior research has suggested that blockade of KKS, in particular PK, can improve acute phase outcomes and decrease infarct volumes after tMCAO [4]. We have shown that a delayed and sustained blockade of PK starting from day 3 improved stroke recovery in tMCAO mice. Administration of αPK reduced inflammation, improved functional outcomes, and ameliorated neurological deficits, accompanied by increased early markers for vessel recovery and vascularization.

Currently, only a few studies have elaborated the long-term role of KKS components in the chronic phase of cerebral ischemia. Longitudinal brain proteome analysis revealed enhanced abundance of kininogen 1 and bradykinin up to 7 days in the ischemic mouse brain [22]. This is in line with our results, where elevated PK and bradykinin concentrations were measured in the blood from day 1 onwards. Other studies have shown significant upregulation of B1R expression during the first 48 h after ischemic stroke in mice and focal brain injury, respectively [23, 24]. We observed increased expression of B1R on vessels at day 7, which was ameliorated by ⍺PK treatment. Among KKS components, PK inhabits a dual mode of action as a proinflammatory and prothrombotic enzyme [25]. In this manner, activation of the KKS has been shown to interact with adhesion molecules and fibrinogen, potentially affecting thrombus formation [26, 27]. Thrombotic activity has been reported to persist for up to 24 h after reperfusion in 60-min tMCAO mice [28, 29]. Furthermore, thrombus formation has been described until day 28 in a photothrombosis model of ischemic stroke [28, 29], although this may not be ideal to mimic the microvascular consequences of human stroke. In addition, thrombi have been found to persist in the microvasculature until day 28 in a 30-min tMCAO model, but subacute depletion of platelets was unable to protect mice from ischemic stroke consequences and failed to improve functional outcome [16]. In contrast, our study demonstrated that administration of αPK antibodies in the subacute phase after tMCAO reduced KKS activation, diminished persistent microvascular thrombosis, and improved functional outcomes at day 7. In light of these results, it is likely that the regenerative effect of PK inhibition is probably not due to the thrombogenic properties of PK but rather mediated through cell-modulatory effects, presumably at the neurovascular unit.

One of the major complications after ischemic stroke is the breakdown of the BBB. Increased permeability follows a multiphasic pattern of an open-close-open phenomenon (∼ 6 h, ∼ 72/96 h, ∼ 1 week) in rodent and human stroke [30]. By blocking PK in our study, the extravasation of albumin and PK into the brain parenchyma was mitigated, indicating stabilization of the BBB after the start of treatment on day 3. Endothelial-stabilizing mechanisms are predominantly mediated by intracellular Rac1 in conjunction with activating RhoA, leading to cytoskeletal rearrangement [21]. Under inflammatory conditions, activation of B1R leads to the formation of myosin light chains and actin stress fibers and phosphorylation of belt-like adherent junctions, causing dissociation of TJ, e.g., ZO and occludins at the endothelial barrier [31]. This process was significantly ameliorated in purified endothelial cells of αPK antibody-treated animals in our study, as indicated by increased Rac1 and decreased ROCK expression, accompanied by increased TJ coverage of vessels in peri-infarct areas. Additionally, ischemic stroke triggers MMP activity, notably MMP-2 and MMP-9, implicated in BBB disruption and extracellular matrix degradation. MMP-9 induces complete basal lamina and TJ breakdown, exacerbating barrier impairment and vasogenic edema post-stroke [32–34]. In our study, subacute administration of αPK reduced MMP-2 and MMP-9 levels and attenuated BBB breakdown and edema formation, supporting recovery after cerebral ischemia. Ang-1 and α5 integrin have been described as subacute angiogenic markers after tMCAO, expressed at the ischemic core border and penumbra [35], and both markers were increased after PK inhibition at day 7. In contrast, VEGF expression increases early after stroke [36] and was not changed by our treatment.

Another consequence of BBB breakdown during cerebral ischemia is an increase in inflammatory processes and leukocyte infiltration. The KKS influences activation of immune cells, their growth, movement, and functions [37]. Moreover, it plays a role in recruiting immune cells from the bloodstream, particularly monocytes and T lymphocytes, as shown in a mouse model of infection [38]. Reduced numbers of infiltrated CD11b^+^ macrophages/microglia and Ly6B.2^+^ neutrophils, as well as reduced expression of inflammatory cytokines, have been observed in PK^−/−^ mice at 24 h after 60-min tMCAO [9]. Interestingly, in experimental encephalomyelitis (an animal model of multiple sclerosis), deficiency or pharmacologic blockade of PK significantly reduced immune cell migration into the brain by modulation of the BBB [39]. We observed a significant reduction in infiltrated CD11b^+^ macrophages/microglia and CD3^+^ T cells in αPK-treated mice. Additionally, cytokines IL-1β, IL-6, and RANTES were significantly reduced in the ischemic brain in αPK-treated mice in our study, underlying the reduction in T cells and macrophages described as major peripheral sources of these cytokines [40].

From a clinical perspective, drugs supporting post-stroke recovery have distinct therapeutic targets that are related to plasticity and neuronal growths after stroke; improvements in behavioral outcomes are not necessarily connected to a reduction in infarct volume [41]. Among the drugs that enhance motor recovery in humans, serotonergic and dopaminergic agents currently represent promising targets. Restorative stroke therapies have generally been targeted at the brain, rather than arterioles, clots, or platelets [41]. However, in our study we describe an intervention window that connects the vascular pathology and the recovery process and both can be targeted by PK inhibition. Therapeutic success and safety for PK inhibition has already been demonstrated in the disease of hereditary angioedema (HAE), which harbors deficiency or dysfunction in the C1-esterase inhibitor that normally inhibits PK and activates FXII [42]. Consequently, PK activity increases blood vessel permeability, allowing fluid to pass through the blood vessel walls and causing edema formation – a process that we also observed in our study during cerebral ischemia. In a permanent model of MCA occlusion in mice, treatment with the human recombinant PK inhibitor DX-88 (approved for HAE) in the acute phase of ischemic stroke reduced infarct volume and brain swelling, lowered the amount of dying neurons, and drastically diminished neurological damage for up to 7 days post-ischemia [43]. Thus, although successful PK inhibition has been approved in other diseases, it has not yet been translated to ischemic stroke therapy.

In summary, we found that inhibition of PK administered 3 days post-stroke reduced infarct volume and improved neurological outcomes, with sustained effects until day 7. Mechanisms supporting the recovery included stabilization of the BBB, a reduction in thrombosis and the inflammatory response, and increased angiogenic signaling.

### Electronic supplementary material

Below is the link to the electronic supplementary material.

Supplementary Material 1

## Data Availability

Data used for this manuscript are available upon reasonable request.
