# Supplementary material for: Delayed plasma kallikrein inhibition fosters post-stroke recovery by reducing thrombo-inflammation
Source: J Neuroinflammation. 2024 Jun 13;21:155. doi: 10.1186/s12974-024-03149-w (PMC11177352; doi:10.1186/s12974-024-03149-w)
Supplement: Supplementary file 1 — Supplementary Material 1 [file 12974_2024_3149_MOESM1_ESM.docx]

# Supplement


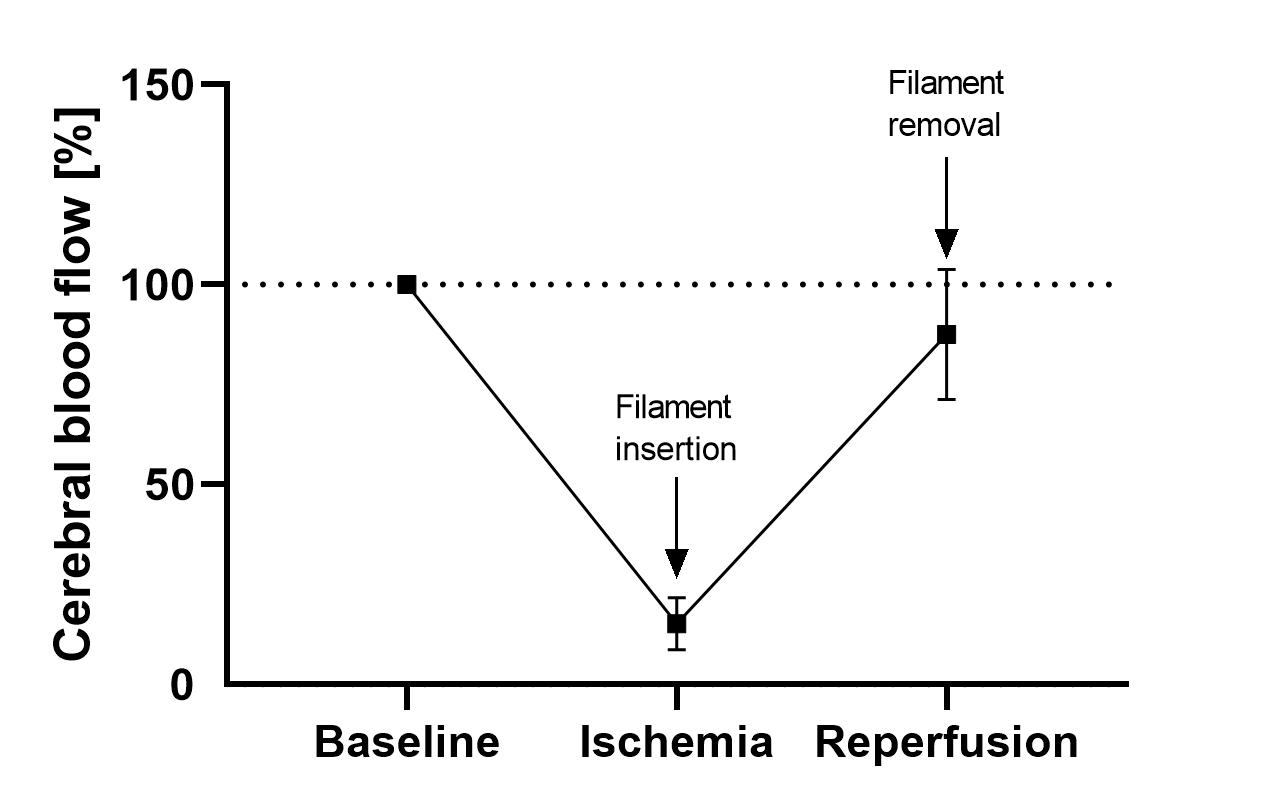


**Suppl. Fig. 1: Measurement of cerebral blood flow during tMCAO.** Determination of cerebral blood flow before ischemia (baseline), immediately after insertion of the filament (ischemia), and after filament removal (reperfusion) by serial Laser Doppler Flowmetry (n=10).


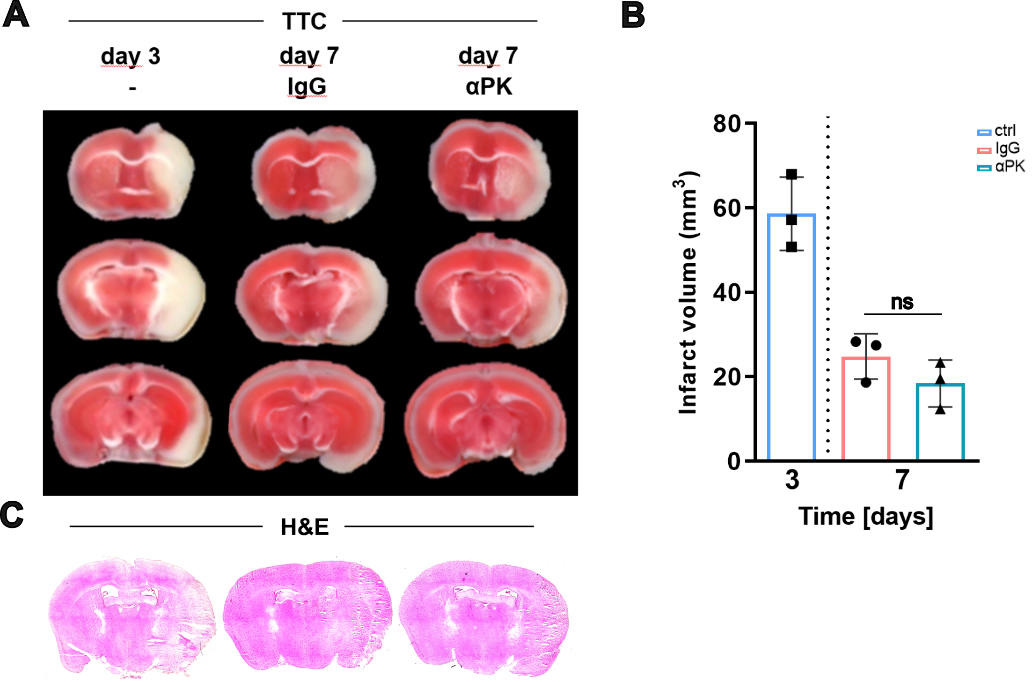


**Suppl. Fig. 2: Histological evaluation of infarct sizes after tMCAO.**  (A) TTC staining of 3 coronal brain sections of day 3 untreated tMCAO mice, of day 7 tMCAO mice with either αPK-treatment or respective control IgG. (B) Visualization of cerebral damage is indicated by the representative and corresponding H&E staining of the infarcted brain. (C) Quantification of infarct volume at indicated time points and groups (n=3 each group). Infarct sizes showed tendential reduction in αPK- compared to IgG-treated mice. Two-way ANOVA and post hoc Sídák test for αPK vs. IgG.


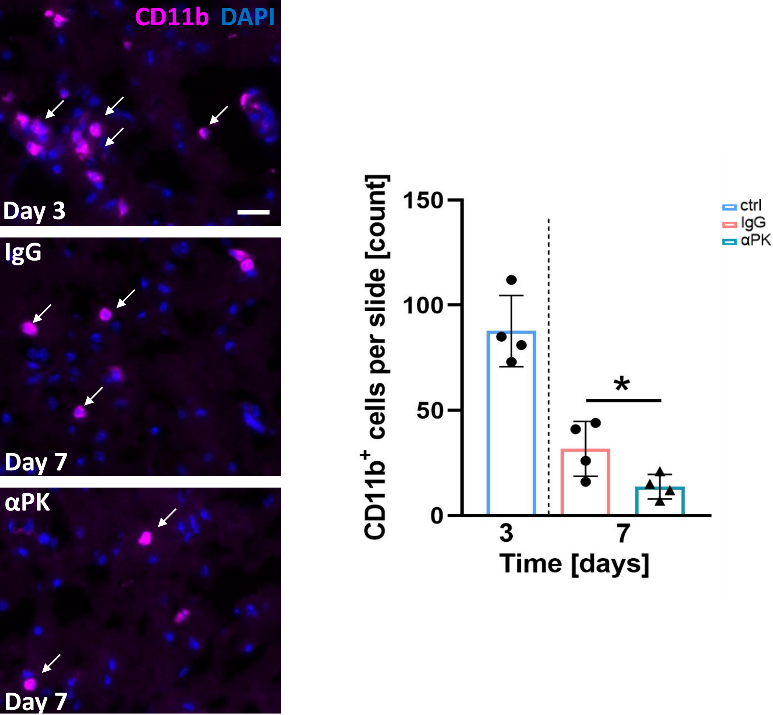


**Suppl. Fig. 3: Histological analysis of infiltrated CD11b^+^ macrophages.** Representative images of CD11b, DAPI macrophage staining of unfixed tissue from day 3, day 7 αPK-treated and day 7 IgG-treated tMCAO mice (n=4 each group). One-way ANOVA and post hoc Dunn's test. **P* < 0.05 for αPK vs. IgG.
